# Training of the trainer for health professionals: sharing experience from Turkey to Tanzania

**DOI:** 10.1080/10872981.2026.2622840

**Published:** 2026-02-02

**Authors:** Aysel Başer, Mustafa Küçük, Ömer Faruk Sönmez, Hakan Gülmez, Funda İfakat Tengiz, Hale Sezer, Gürkan Yıldız, Seçil Arslansoylu Çamlar, Said Salum Kilindimo, Hatice Şahin

**Affiliations:** a Izmir Demokrasi University, Faculty of Medicine, Department of Medical Education, Izmir, Türkiye; b Uşak Provincial Health Directorate, General Directorate of Health Services, Uşak, Türkiye; c School of Medicine and Population Health, University of Sheffield, Sheffield, UK; d Vocational School of Health Services, Kırıkkale University, Kırıkkale, Türkiye; e Izmir Demokrasi University, Faculty of Medicine, Department of Family Medicine, Izmir, Türkiye; f Izmir Katip Çelebi University, Faculty of Medicine, Department of Medical Education, Izmir, Türkiye; g İzmir Bakırçay University, Faculty of Health Sciences, Department of Nursing Education, Izmir, Türkiye; h Buca Seyfi Demirsoy Training and Research Hospital, Orthopedics and Traumatology Clinic, Izmir, Türkiye; i Pediatric Nephrology Clinic, Faculty of Medicine, University of Health Sciences, Izmir, Türkiye; j Emergency Medicine Department, Muhimbili University of Health and Allied Sciences, Dar es salaam, Tanzania; k Ege University, Faculty of Medicine, Department of Medical Education, Izmir, Türkiye

**Keywords:** Train-the-trainer, disaster management, health professionals, leadership, emergency medicine, medical education

## Abstract

**Background:**

Effective disaster management requires healthcare professionals to function not only as responders but also as trainers who can disseminate knowledge and skills. In low-resource settings such as Tanzania, structured train-the-trainer (ToT) programs tailored to physicians remain limited. This study aimed to design, implement, and evaluate a trainer development program for Tanzanian emergency medicine physicians using the ADDIE instructional design framework to transparently link needs assessment to role-adapted objectives, learning activities, and evaluation.

**Methods:**

A mixed-methods design was applied, combining pre-training surveys, post-training assessments, and thematic feedback analysis. The program, conducted on 21–22 November 2024 at the Urla International Emergency Disaster Training and Simulation Centre, followed the ADDIE model (Analysis, Design, Development, Implementation, Evaluation). Twenty Tanzanian physicians (mean age 35.9 years, 60% female, mean experience 8.2 years) participated. Participants were grouped as Emergency Medicine Specialists/Medical Officers (n=8) and General Practitioners/Resident Physicians (n=12), with tailored objectives focusing on leadership, teamwork, disaster planning, and trainer skills.

**Results:**

Participants achieved a mean post-training MCQ score of 17.68 out of 20, corresponding to an overall correct response rate of 88.4%. Scenario-based and interactive learning methods were highly valued, while insufficient training duration and limited technical infrastructure were identified as challenges. Emergency medicine specialists prioritized leadership and coordination skills, whereas general practitioners and residents emphasized educational strategies and program development.

The program was feasible and well received, and participants achieved high immediate post-course knowledge scores and reported strong perceived value of scenario-based and trainer-focused learning activities. The findings support role-adapted ToT models for physicians; however, objective measurement of educator and leadership competencies and follow-up assessment of cascade training implementation are needed to determine sustained trainer development.

## Background

Effective management of health services in disaster situations requires a multidisciplinary approach. In this context, training health workers and enhancing their leadership capacity in disaster management play a critical role in increasing the resilience of healthcare systems. Trainer development programmes provide an important platform to ensure the transfer of both knowledge and practical skills [1–3].

In the evolving landscape of disaster management education, training-of the-trainer (ToT) programmes have gained significant attention as an effective strategy for enhancing the capacity of local trainers, particularly in resource-limited settings. This model ensures the sustainability of training efforts by equipping local experts who, in turn, disseminate knowledge within their communities, thereby creating a multiplier effect [4]. Unlike traditional training programmes that rely heavily on external experts, ToT programmes foster regional collaboration, integrating healthcare professionals, emergency responders, and policymakers to strengthen multidisciplinary disaster response systems [4,5].

Despite the increasing adoption of the ToT model, existing literature reveals several critical gaps. Many ToT programmes in healthcare focus primarily on technical competencies related to clinical skills, disaster triage, and emergency medical procedures [6,7]. However, limited emphasis is placed on developing the educational and leadership skills of healthcare professionals to function effectively as both trainers and mentors [7]. The ability to train others requires not only subject matter expertise but also instructional methodologies, curriculum development skills, and pedagogical proficiency, which are often overlooked in disaster preparedness programmes. This gap raises concerns about the long-term sustainability of disaster training initiatives and their effectiveness in building local resilience.

Another key limitation in the literature is the lack of structured TOT programmes that are specifically designed for physicians. While ToT models have been successfully implemented in nursing, paramedic, and community health training programmes, the role of physicians as educators in disaster management remains underexplored [3,8]. Physicians are often expected to lead disaster response teams, yet few training programmes provide them with the necessary tools to become effective educators within their institutions. As a result, knowledge transfer remains inconsistent, and many healthcare professionals struggle to sustain a culture of continuous education and preparedness in their respective healthcare systems [7].

Moreover, emerging challenges such as the COVID-19 pandemic have further reshaped disaster training methodologies, highlighting the need for flexible and scalable educational models [2]. While virtual platforms have expanded access to disaster training, their integration into structured ToT programmes remains limited, particularly in low-resource settings where technological and logistical barriers persist [2]. There is a growing need to bridge traditional in-person training with innovative digital approaches to ensure wider accessibility and continuous skill development in disaster preparedness education.

Train-the-trainer models are widely used to disseminate knowledge and skills in health professions education, but systematic reviews have shown substantial heterogeneity in programme content and delivery methods, with evaluation commonly emphasising short-term outcomes and variable evidence for sustained downstream impact [8–11]. In resource-limited settings, ToT approaches are frequently adopted as a pragmatic strategy for scaling emergency and preparedness education, yet published evaluations often highlight persistent challenges in demonstrating long-term sustainability and effects beyond immediate learning [10–12]. Consistent with calls to strengthen disaster education through pre-training gap analyses and competency-informed curriculum development [13], the present programme was designed explicitly as a trainer-development intervention for physicians, embedding educator competencies (adult learning principles, teaching strategies, assessment literacy, multiple-choice question construction, and programme evaluation) alongside leadership and interprofessional collaboration, and reinforcing these through scenario-based learning aligned with disaster types relevant to Tanzania. In addition, objectives and learning activities were tailored by professional role based on the pre-training needs assessment rather than assuming a uniform one-size-fits-all ToT approach [13].

Conceptually, the programme was guided by an adult-learning and capacity-building logic model in which trainer development is expected to occur through relevance-driven, problem-centred learning activities supported by interactive practice and feedback, and to translate into organisational capacity via cascade training. In this framework, educator development is operationalised as acquisition and intended use of educational design and delivery competencies such as learner-centred teaching strategies, assessment literacy, and programme evaluation, whereas leadership development is operationalised as skills applied in team-based learning and facilitation, including communication, coordination, and collaborative decision-making during scenario work. The evaluation was designed to capture short-term outcomes aligned with this model, focusing on immediate learning and perceived applicability rather than confirmed behavioural change or institutional adoption.

This study addresses these gaps in the literature by designing and evaluating a structured TOT programme tailored specifically for Tanzanian emergency physicians involved in disaster and emergency management. Unlike many existing ToT programmes, which focus solely on technical skills, this study integrates leadership development, educational methodology, and scenario-based pedagogical training, ensuring that participants not only gain knowledge but also acquire the competencies to train others effectively.

This study contributes to the ToT literature in disaster and emergency management education by reporting a physician-focused trainer development programme that intentionally embeds educator competencies alongside leadership and interprofessional collaboration, responding to prior reviews that note wide variation in ToT design and limited clarity on which delivery characteristics best support robust outcomes [8,9]. By documenting the linkage from needs assessment to role-adapted objectives, learning activities, and evaluation, the study provides a replicable structure for iterative improvement in contexts where ToT models are frequently used but sustainability and practice-level outcomes are not consistently demonstrated [10–12]. Finally, the needs-assessment–driven role adaptation aligns with recommendations that disaster education should be competency-informed and calibrated to differing professional baselines rather than delivered as undifferentiated training.

The ADDIE model is a well-established instructional design framework used to structure training development from analysis through evaluation, and its use here is intended to increase transparency and replicability rather than to claim methodological novelty. The novel element of this study is the development of a physician-focused ToT that was adapted to Tanzanian emergency physicians through a pre-training needs assessment and explicit differentiation by professional role, responding to published ToT reviews that highlight wide variability in programme design and the importance of specifying which design features are expected to drive outcomes [14].

This trainer development programme was implemented within an established cooperation track between Turkish and Tanzanian institutions aimed at strengthening emergency and disaster preparedness through capacity building and knowledge transfer. In Tanzania, prior ‘Introduction to Disaster and Emergency Response (ADG+)’ activities were delivered in cooperation with the Ministry of Health of Tanzania with involvement of Muhimbili University of Health and Allied Sciences (MUHAS), reflecting a pre-existing national academic and service platform for emergency medicine training and workforce development [15,16]. From the Turkish side, the initiative aligns with Türkiye’s broader practice of conducting international health trainings through the Ministry of Health and its external cooperation mechanisms, including structured training programmes coordinated through its foreign affairs/eu cooperation directorate and implemented with partner countries [17]. This specific ToT course in İzmir was therefore positioned as a sustainability step within ongoing emergency medicine capacity-building efforts, aiming to develop local physician-trainers who could support cascade dissemination after returning to Tanzania, rather than as a one-off training event.

## Methods

### Study design

This study employed a mixed-methods research design to comprehensively evaluate a train-the-trainer programme for health professionals. Quantitative data were collected using pre-training questionnaires to assess participants’ expectations and post-training assessments to capture immediate learning outcomes. Qualitative data were obtained from participants’ anonymised written responses to structured open-ended questions included in the post-training feedback form, aimed at exploring participant experiences and perceived strengths and areas for improvement of the programme.

### Participants

Eligibility criteria were Tanzanian physicians affiliated with emergency care (Emergency Medicine Specialists/Medical Officers, General Practitioners, or Resident Physicians) who were nominated by their institutions and available on 21–22 November 2024. Exclusion criteria were inability to attend both training days or lack of consent. Recruitment was coordinated via the Muhimbili University of Health and Allied Sciences and partner institutions; all invited physicians who consented were enroled. There were no missing data for the MCQ or feedback forms; analyses were complete-case.

#### Programme design (ADDIE model)

The ADDIE instructional design model (Analysis, Design, Development, Implementation, Evaluation) was applied to structure programme development and document the link between needs, objectives, training activities, and evaluation [14]. In this study, ADDIE is used as an organising framework; the novelty lies in its application to create a needs-assessment–driven, role-adapted ToT for Tanzanian emergency physicians rather than in the ADDIE framework itself. The model consists of five key phases: Analysis, Design, Development, Implementation, and Evaluation, ensuring that the programme was tailored to the needs of Tanzanian emergency physicians and aligned with best practices in disaster management education Intervention description and reporting were structured to align with the template for intervention description and replicaton (TIDieR) checklist to improve transparency and replicability of the training intervention [18].

1. **Problem Identification and Needs Analysis (Analysis Phase)**

This programme was designed as a continuation of the Emergency Medicine Capacity Building Programme, coordinated by the Turkish Ministry of Health’s Directorate General for European Union and Foreign Affairs in collaboration with the Turkish Cooperation and Coordination Agency (TİKA). The primary objective of the programme was to strengthen healthcare systems in Africa by enhancing disaster preparedness and emergency response capacities.

The choice of Tanzania as the recipient context was driven by an existing partner network and an identified need to strengthen emergency and disaster preparedness capacity through physician-led training, supported by Tanzanian institutional stakeholders. Previous ADG+ training activities in Dar es Salaam involved Tanzanian Ministry of Health representation and MUHAS participation, and Tanzanian partners nominated participants for this ToT as part of a pathway toward local trainer capacity [15] discussion. The choice of Türkiye as the source context reflected the availability of established emergency medicine training expertise and simulation-based training infrastructure, and the course was delivered within Türkiye’s broader international health cooperation practice in which structured trainings are coordinated through the Ministry of Health’s international cooperation directorate.

The initiative was specifically tailored to help participating physicians better understand pedagogical training processes, enhance interprofessional collaboration, and develop leadership skills essential for effective disaster and emergency management.

The needs analysis phase aimed to identify the training gaps and specific learning needs of Tanzanian emergency physicians in disaster and emergency management. To achieve this:

- Open-ended questions were used in a preliminary survey to gather participants' expectations and perceived training needs.
- Consultations were held with emergency medicine experts, local healthcare administrators, and previous trainees to refine the core competencies required for the programme.
- Key focus areas identified included leadership in crisis situations, teamwork in multidisciplinary settings, disaster response strategies, and effective educational methodologies.

2.
**Curriculum and Content Development (Design Phase)**

Following the needs assessment, the curriculum was structured to align with adult learning principles, interprofessional collaboration, and leadership development, and the detailed course schedule is provided in the Supplementary Material.

**Figure 1.**
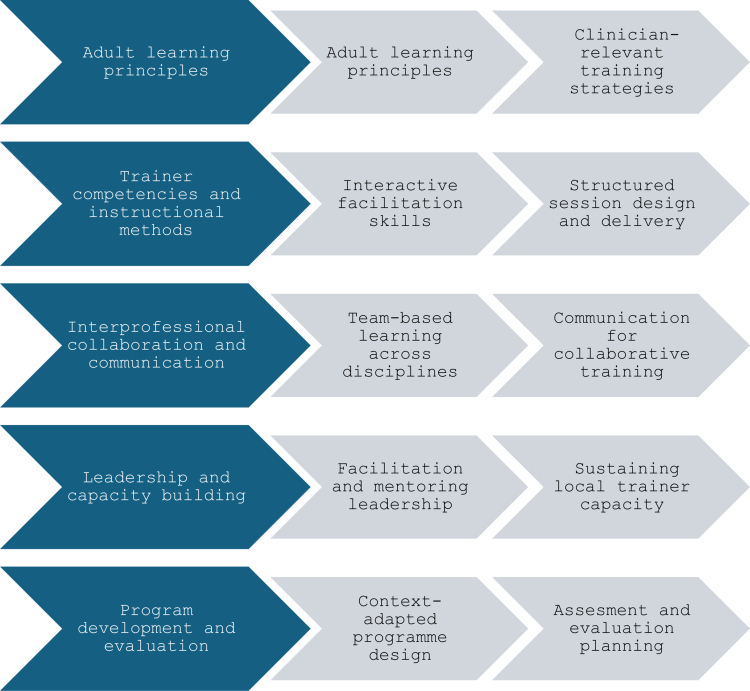
Trainer development curriculum objective framework (Design phase).

In the Design phase, the curriculum was developed using the needs assessment to align learning objectives with participants’ professional roles and to integrate adult learning principles with scenario-based and interactive approaches. Two trainer planning meetings were held to finalise content sequencing and instructional methods, and the resulting five-domain objective framework is summarised in Figure 1.

3.
**Programme Development and Structuring (Development Phase)**

The development phase focused on refining the training materials and assessment tools. All lecture content, scenarios, and supporting materials were compiled in a shared cloud-based repository to enable iterative peer review, harmonisation across instructors, and final revisions prior to delivery. The course structure was finalised as a blended format combining brief didactic inputs with simulation-based exercises and small-group discussion to reinforce application, feedback, and peer learning. A post-training MCQ aligned with the programme objectives and a structured feedback form were developed to capture immediate learning outcomes and participant perceptions.

4.
**Implementation of the Training Programme (Implementation Phase)**

The two-day programme was delivered on 21–22 November 2024 at the Urla International Emergency Disaster Training and Simulation Centre with 20 Tanzanian physicians. Delivery emphasised experiential learning through disaster simulations, role-playing, and guided debriefings, supported by facilitated discussions that explicitly linked theoretical concepts to hands-on practice. For selected activities, participants were grouped by professional background (Emergency Medicine Specialists/Medical Officers versus General Practitioners/Resident Physicians) to support role-relevant engagement with the learning objectives.

5.
**Programme Evaluation and Outcome Assessment (Evaluation Phase)**

The evaluation phase was designed to capture short-term outcomes aligned with the programme’s conceptual framework, focusing on immediate learning and perceived applicability of trainer-development content. A mixed set of quantitative and qualitative outcomes was selected to reflect both knowledge acquisition and participant experiences following the training.

Our evaluation focused on immediate outcomes and should be interpreted accordingly. Participant feedback corresponds to short-term reaction outcomes, and the MCQ corresponds to short-term learning outcomes; we did not directly measure trainer identity, teaching competence, or post-course training behaviours (e.g. observed teaching performance, cascade trainings delivered) and therefore cannot claim behaviour change or sustained trainer capacity on the basis of these data alone. This limitation is consistent with common patterns in the train-the-trainer literature, where evaluations frequently emphasise immediate learning outcomes and heterogeneous measures, and where evidence for sustainability and downstream effects is less consistently demonstrated [18]. A follow-up evaluation at 3 and 6 months is planned to document cascade trainings delivered, reach, and implementation barriers, and to assess trainer practices using brief self-report and structured observation where feasible. Assessment Tools:

The primary quantitative outcome was immediate post-training knowledge, assessed using a 20-item multiple-choice questionnaire (MCQ; score range 0–20, reported as percentage correct). The MCQ was developed collaboratively by the training team and blueprint-mapped to the programme’s trainer-development learning objectives, including adult learning principles, teaching strategies, assessment literacy, and programme evaluation. The assessment was intended as a process-oriented formative measure within a pilot educational intervention and was not designed for summative or high-stakes use.

Qualitative outcomes were derived from structured open-ended questions included in the post-training feedback form, which invited participants to describe strengths of the programme and areas for improvement. The same assessment tools and administration procedures were used for all participants.

### Statistical analysis

#### Quantitative data

Quantitative data were analysed using descriptive statistics. Participant characteristics, pre-training expectations, and post-training MCQ results were summarised using frequencies, percentages, means, and standard deviations, as appropriate. Given the pilot nature of the study and the small sample size, no inferential statistical analyses were performed.

#### Qualitative data

Qualitative data from written feedback responses were analysed using inductive thematic analysis. Four independent coders with formal training in qualitative research and expertise in health professions education conducted parallel coding of the full dataset. Two structured consensus meetings were held to compare codes, resolve discrepancies, and agree on final themes. Formal inter-rater reliability statistics were not calculated due to the exploratory nature of the study; instead, analytic rigour was ensured through independent parallel coding, investigator triangulation, and consensus-based theme development. Representative quotations were selected to illustrate each theme.

All coders independently reviewed and coded the complete dataset. Two structured consensus meetings were subsequently held to compare codes, resolve discrepancies, and refine codes into final themes. Given the exploratory and pilot nature of the study, formal inter-rater reliability statistics were not calculated; instead, analytic rigour was ensured through independent parallel coding, investigator triangulation, and consensus-based theme development. Representative quotations were selected to illustrate each theme.

## Results

Results are presented as (i) needs-assessment findings and participant characteristics, (ii) immediate post-course learning outcomes based on the end-of-course MCQ, and (iii) post-course thematic findings from participant feedback. Consistent with our evaluation scope, these findings reflect short-term learning and perceived applicability rather than observed teaching performance, leadership competence, or verified cascade training implementation [18].

1.
**Problem Identification and Needs Analysis (Analysis Phase)**

The average age of the participants was 35.9 years (range 28-51 years), 60% were female (*n* = 12) and 40% were male (*n* = 8).

A qualitative data analysis was conducted to identify the expectations, needs, and learning priorities of the participants based on their professional backgrounds and prior experience in disaster and emergency management.

### Prior experience and active participation in disaster management

A subset of participants shared their previous experiences in disaster management:

- Active Participants (*n* = 6): Engaged in real-life disaster response efforts, such as fire accidents and motor vehicle crash response.

*“I was part of a medical team deployed to assist fire victims.”*

- Disaster Planning and Preparedness (*n* = 4): Assisted in hospital disaster planning and drills.

*“I participated in the hospital disaster response plan and took part in two drills.”*

- Training and Awareness Initiatives (*n* = 2): Took part in educational and awareness programmes about disaster response.

*“I am a hospital disaster response trainer and part of the community first aid training team.”*

- No Previous Experience (*n* = 4): Some participants had no prior real-life exposure to disasters.

*“I haven’t participated in any disaster response efforts so far.”*

The following table presents the themes, sub-themes, and codes derived from the qualitative analysis of participant expectations (Table 1). The findings indicate that most participants prioritised professional disaster management skills over educator competencies, despite the programme's focus on trainer development.

**Table 1.** Coding of participant expectations and needs.

Theme	Sub-theme	Code (n:)	Example response
Disaster and emergency response training (66.6%)	Disaster preparedness	Learning how to respond effectively in disasters, Disaster preparedness and Response	‘I want to learn how to manage disasters in different settings with limited resources.’
	Equipment and resource utilisation	Understanding the use of medical equipment in disasters, Resource Utilisation	‘I want to see Turkey’s advanced emergency response systems and how they operate.’
	Coordination & teamwork	Improving collaboration in emergency settings, Teams, Coordinate,	‘I want to learn how teams can be effectively coordinated during disasters.’
Trainer development and educational skills (33.3%)	Educator competencies	Becoming a better trainer, Peer education,	‘I want to become a better trainer so I can educate my colleagues more effectively.’
	Teaching strategies	Learning interactive, teaching methods, Effective Teaching Strategies	‘I need to learn more about interactive training strategies and methodologies.’
	Training evaluation	Measuring trainee learning outcomes	‘I need to know how to evaluate whether my trainees have truly learned the material.’

The needs analysis also identified a clear expectation–objective mismatch at baseline. Although the course was designed primarily as a trainer development programme emphasising educator competencies, most participants initially described their primary learning goals in terms of technical disaster response skills and systems exposure. This mismatch is an important finding because it shaped how participants interpreted the course and it constrains the strength of claims that educator roles were ‘strengthened,’ which in this study can only be supported by immediate post-course feedback rather than direct assessment of teaching performance or post-course trainer behaviours.

### Findings from the analysis phase in ADDIE programme design

Analysis-phase findings indicated two distinct expectation profiles by professional role. Emergency medicine specialists and medical officers (*n* = 8) most frequently emphasised leadership, teamwork, and coordination in disaster settings, whereas general practitioners and residents (*n* = 12) more often highlighted trainer-oriented needs such as teaching strategies, programme development, and evaluation. Overall, most participants framed their primary expectations around technical disaster response rather than trainer development (66.6% vs 33.3%), indicating an expectation–objective mismatch at baseline. Participants also expressed a strong preference for practical, scenario-based learning and for exposure to established disaster response systems, which informed the selection of simulation and applied activities in the subsequent design phase.

2.
**Curriculum and Content Development (Design Phase)**

Following the needs assessment phase, where participants’ expectations and training gaps were identified, the training curriculum was structured to align with adult learning principles, interprofessional collaboration, and leadership development. The trainers were informed about the programme objectives, learning outcomes, and instructional methodologies, ensuring that the course effectively addressed both trainer development and disaster preparedness education. Based on these considerations, the finalised course structure was designed as shown in Supplementary Material.

3.
**Programme Development and Structuring (Development Phase)**

In the Development Phase of the ADDIE model, the training materials, theoretical lecture presentations, disaster scenarios, small group activities, data collection forms, and assessment tools were designed and structured by the instructors. A collaborative approach was adopted, ensuring that all educational components were aligned with the learning objectives identified in the analysis phase. To assess participants' knowledge acquisition, 20 multiple-choice questions (MCQs) were developed based on the learning objectives. These questions underwent technical review and validation by other trainers, ensuring clarity, relevance, and alignment with the educational goals. Additionally, peer-review sessions were conducted to refine the scenario-based exercises, evaluation forms, and interactive teaching strategies, enhancing the overall quality and effectiveness of the training programme.

4.
**Implementation of the Training Programme (Implementation Phase)**

The overall post-training MCQ performance was high, with a mean score corresponding to an overall correct response rate of 88.4%.Participants answered an average of 17.68 out of 20 questions correctly, while the average number of incorrect responses was 2.32. Although the overall test comprehension was strong, some participants struggled with specific questions (notably Questions 5, 6, and 7). The low number of unanswered questions suggests that the test items were generally well-understood. A noteworthy finding is that the most challenging questions were exclusively related to lecture-based theoretical sessions, emphasising presentation-based learning objectives. This suggests that participants may have found practical, scenario-based content more comprehensible and retainable compared to purely theoretical instruction. However, item analysis indicated that the topics covered in the most challenging questions may need to be reviewed and reinforced in future training sessions to improve comprehension and retention (Figure 1 in Supplementary Material).

The MCQ was blueprint-mapped to the programme’s trainer-development objectives rather than to clinical disaster medicine competencies. Items primarily assessed adult learning principles, teaching methods, assessment and evaluation, and programme development content delivered across Day 1 and Day 2, with scenarios used as applied contexts for instructional practice. The lowest-performing items were mapped to lecture-delivered theoretical content, whereas content reinforced through interactive activities and scenario-based discussions showed higher performance in the immediate post-course assessment.

5.
**Programme Evaluation and Outcome Assessment (Evaluation Phase)**

In the Evaluation Phase of the ADDIE model, qualitative data analysis was conducted to assess both the strengths and areas for improvement in the training programme. Participant feedback was categorised into positive aspects and suggested improvements, which were systematically analysed to enhance future training sessions (Table 2).

**Table 2.** Programme evaluation findings.

Theme	Sub-theme	Code	Example response
Strengths of the programme	Content & organisation	Well-structured curriculum	‘The training was well-structured and provided extensive knowledge relevant to our professional needs.’
		Alignment with professional needs	‘The topics covered were directly applicable to my role in disaster response.’
	Interactive learning	Engaging discussions and teamwork	‘The interactive format helped us engage with both instructors and peers.’
		Active participation encouraged	‘I appreciated how we could ask questions and share experiences.’
	Effective teaching strategies	Scenario-based learning	‘The scenario-based learning and assessment preparation were particularly useful.’
		Use of multiple teaching techniques	‘The combination of lectures, discussions, and group work made learning more effective.’
	International perspective	Exposure to global practices	‘Learning about Turkey’s health system and disaster preparedness strategies was insightful.’
		Networking opportunities	‘Meeting professionals from different countries was valuable.’
Areas for Improvement	Time management	Training duration too short	‘The duration was too short; more time should be allocated to practical exercises.’
		Need for more in-depth sessions	‘Some topics needed more time to be fully explored.’
	Language barriers	Communication challenges	‘Some instructors should improve their language proficiency for better communication.’
		Need for translation support	‘Providing translations for key concepts would help understanding.’
	Technical & internet issues	Connectivity problems	‘There were occasional difficulties in accessing online materials during the training.’
		Need for digital resources	‘More pre-recorded sessions or online materials would be useful.’
	Training materials & technology use	Diversifying instructional materials	‘More diverse instructional materials and technological tools would enhance learning.’
		Incorporating digital tools	‘Using interactive software for assessments would be beneficial.’
	Hands-on training & scenario expansion	More real-case simulations	‘We need more hands-on activities and expanded real-case disaster scenarios.’
		Practical application of knowledge	‘More time for scenario-based exercises would help reinforce learning.’
	Additional social & networking activities	Including cultural and networking opportunities	‘Including cultural or networking activities would enhance collaboration among participants.’
		More informal engagement sessions	‘Having informal meetups with trainers and peers would strengthen connections.’

Participant reflections also provided direct qualitative evidence of trainer-oriented learning and leadership-related experiences. In the ‘Strengths’ themes, participants repeatedly emphasised the usefulness of scenario-based learning and assessment preparation, and they described active engagement, teamwork, and the opportunity to practice interactive teaching approaches. In the ‘Areas for improvement’ themes, participants requested more time for hands-on and scenario-based practice and suggested strengthening technical infrastructure to support technology-enhanced teaching, indicating that participants recognised trainer-relevant components but sought more opportunities for coached application.

Consistent with the conceptual framework, educator-development themes were reflected in participants’ emphasis on teaching strategies, assessment preparation, and programme evaluation, while leadership-related themes were reflected in comments on teamwork, engagement, communication, and coordination during group discussions and scenario debriefings. These qualitative findings indicate perceived applicability of educator and leadership components, although objective competence and post-course cascade implementation were not measured.

## Discussion

The trainer development programme implemented in this study was designed to strengthen the educator roles of Tanzanian healthcare professionals in disaster management. The findings revealed that the majority of participants focused on technical skills related to disaster management, while less emphasis was placed on developing educator roles and pedagogical competencies. The findings also indicated that different professional groups had distinct priorities and needs. While Emergency Medicine Specialists and Specialist Physicians concentrated more on technical and leadership skills, General Practitioners and Resident Physicians prioritised training and disaster planning at the local level. These differences suggest that train-the-trainer programmes should be tailored to the target audience. Because educator development and leadership competence were not directly assessed with validated instruments or observed performance measures, the discussion interprets leadership and educator outcomes as immediate learning and perceived applicability rather than demonstrated competency gains.

Our item-level findings showed lower performance on questions mapped primarily to lecture-based, theoretical sessions, whereas scenario-based and interactive content appeared more readily learned in the immediate post-course assessment. One plausible explanation is cognitive load. Disaster education and educational-methodology concepts can carry high intrinsic load for clinicians who are simultaneously processing new terminology, unfamiliar assessment concepts, and a new learning environment; when working memory capacity is exceeded, learning and performance are impaired, particularly in lecture-heavy formats with limited opportunities for guided practice [19]. In contrast, scenario-based activities may have supported learning through contextualisation, elaboration, and immediate application, which can reduce extraneous load and increase germane processing, particularly for adult learners who prioritise relevance and problem-centred learning [20]. These patterns also suggest that the theoretical components may have benefited from more ‘test-enhanced’ and spaced reinforcement rather than one-time exposure, because distributed practice and retrieval practice have demonstrated benefits for learning outcomes in health professions education and are increasingly recommended as evidence-based strategies to support durable knowledge [21,22]. Accordingly, the lower performance on lecture-mapped items should not be interpreted as evidence that theoretical content is unimportant; rather, it likely reflects modifiable instructional design issues (time on task, opportunities for retrieval, and scaffolding of complex concepts) that are well described in the learning-sciences and medical education literature.

Practically, this can be addressed within resource constraints by embedding brief retrieval opportunities into theoretical sessions (for example, low-stakes single-best-answer questions with facilitated explanation, short recap quizzes at the end of each module, and structured debrief questions that explicitly link scenarios back to underlying principles), and by providing pre-course micro-materials that introduce key terminology so that in-class time can be used for application and feedback. Evidence syntheses in health professions education support both spaced learning approaches and retrieval practice as means to strengthen learning beyond massed, single-session exposure [21].

The literature highlights that trainer development programmes typically focus on enhancing the professional development of healthcare workers, but they often lack sufficient emphasis on pedagogical knowledge and skills necessary for trainers [4]. For instance, Hedberg et al. ’s train-the-trainer programme for chemical and nuclear disaster preparedness primarily focused on technical skill development, but it had limited contributions to the acquisition of teaching competencies. Similarly, Quah et al. conducted a disaster medicine training programme in Nepal that aimed to enhance the clinical response capabilities of local healthcare workers [21]. While the programme supported field-based applications, it lacked a structured approach to developing teaching skills among participants.

A similar pattern was observed in our study, where participants exhibited a stronger interest in acquiring technical knowledge rather than improving their educator identity. This underscores the need for trainer development programmes to focus not only on professional knowledge but also on teaching methodologies and educator roles. While addressing participants' primary learning needs is essential, the findings also suggest that adult learners may not always be fully aware of their educational needs, reinforcing the necessity of a balanced and structured approach to train-the-trainer programmes.

Moreover, Baessler et al. emphasised that interactive learning methods focusing on pedagogical skill acquisition can enhance the effectiveness of train-the-trainer programmes. In our study, participants reported that interactive and scenario-based applied learning processes were particularly beneficial, which aligns with previous findings that trainer development programmes should be structured based on adult learning principles [2,23]. Additionally, a key finding in our study was that participants' exam performance was lower on theoretical, lecture-based learning objectives, while higher on practical, applied learning objectives. This finding is consistent with the literature indicating that interactive and learner-centred approaches are preferred in adult education and are associated with stronger immediate learning outcomes.Our findings further reinforce that trainer development programmes should be designed in alignment with adult learning principles. Tanzanian physicians in our study particularly emphasised the contribution of scenario-based applications to their learning process. This aligns with Hedberg et al.'s findings, which demonstrated that interactive learning methods improve the effectiveness of train-the-trainer programmes [4]. Similarly, Silsbury et al. conducted a train-the-trainer programme in South Wales and found that interactive and practical training models significantly improved trainer development [24].

However, some limitations were identified in our programme, including the insufficient duration of the training and technical infrastructure deficiencies as reported by participants. The literature also highlights that adequate programme duration and well-planned technical resources are essential for maximising the effectiveness of trainer development programmes [4,25,26] also noted that the limited duration of trainer development programmes may prevent certain topics from being thoroughly covered.

Although formal inter-rater reliability statistics were not calculated, the systematic use of independent coding, consensus meetings, and investigator triangulation supports the credibility of the qualitative findings. Considering these factors, future programmes should incorporate extended training periods and improved infrastructure to optimise their impact.

## Conclusion

This training-of-the-trainer programme was feasible and well received among Tanzanian emergency physicians and produced strong immediate post-course knowledge performance. Participant feedback highlighted perceived value of scenario-based and interactive learning, including opportunities to engage in teamwork, discuss implementation challenges, and practice trainer-relevant processes such as assessment preparation and programme evaluation. At baseline, however, most participants prioritised technical disaster-response learning goals over trainer development, indicating an expectation–objective mismatch that limits the strength of inferences regarding educator-role development from immediate post-course data alone. Consistent with our evaluation scope, these findings should be interpreted as evidence of short-term learning and perceived applicability rather than demonstrated teaching competence, leadership competence, or verified cascade delivery.

Participants described intention to adapt elements of the training within their institutions; however, we did not collect follow-up data and therefore cannot provide direct evidence that cascade trainings occurred or that trainer networks were established. To evaluate replication and sustainability, we propose a pragmatic follow-up plan in which participants will be contacted after return to Tanzania at 3 and 6 months to document whether they delivered cascade trainings, the number and professional profile of trainees reached, the content and format delivered, and barriers and enablers to implementation. Where feasible, trainer development will be assessed using brief self-report measures focused on training confidence and teaching practices, combined with documentation of training materials used and a short structured observation checklist during a sample session delivered by a subset of participants. Network development will be assessed descriptively by recording whether participants maintained trainer-to-trainer communication, shared materials across institutions, or received local institutional support for ongoing training activities. This evaluation approach will allow assessment beyond immediate outcomes and provide concrete evidence about replication, reach, and sustainability.

Future iterations should also address the constraints identified by participants, particularly limited time for coached practice and technical infrastructure barriers, by shifting selected theoretical elements to pre-course micro-materials and using in-person time primarily for application, feedback, and scenario-based facilitation practice. In resource-limited contexts, role-adapted trainer development programmes may contribute to preparedness capacity, but their longer-term value depends on demonstrable cascade implementation and sustained institutional uptake.

## Supplementary Material

TOT_Supplementary Material.docx TOT_Supplementary Material.docx

## Data Availability

The datasets generated and analysed during the current study are not publicly available due to participant confidentiality but are available from the corresponding author on reasonable request.
