# Supplementary material for: Training of the trainer for health professionals: sharing experience from Turkey to Tanzania
Source: Med Educ Online. 2026 Feb 2;31(1):2622840. doi: 10.1080/10872981.2026.2622840 (PMC12872079; doi:10.1080/10872981.2026.2622840)
Supplement: TOT_Supplementary Material.docx [file ZMEO_A_2622840_SM0311.docx]

| **Introduction to Disaster and Emergency Response (ADG +) Training of Trainers** | | |  |
| --- | --- | --- | --- |
|  |  |  |  |
| **21.11.2024** | **OPENING AND BASIC TRAINER DEVELOPMENT - DAY 1** | |  |
|  | **LECTURE** | **INSTRUCTOR** |  |
| 09:00-09:30 | Opening | Dr. Ali Özbudak  Prof. Dr. Hatice Şahin – Assoc. Prof. Dr. Aysel Başer- Dr. Mustafa Küçük |  |
| 09:30-10:10 | Overview of the Turkish Health System | Dr. Ali Özbudak |  |
| 10:10-10:40 | Introduction, Gathering Expectations | Prof. Dr. Hatice Şahin – Assoc. Prof. Dr. Aysel Başer |  |
| 10:40-11:10 | Program Overview | Assoc. Prof. Dr. Aysel Başer |  |
| 11:10-11:20 | **COFFEE BREAK** | |  |
| 11:20-11:50 | Interprofessional Education and Collaboration | Assoc. Prof. Dr. Aysel Başer |  |
| 11:50-12:15 | Adult Education | Assoc. Prof. Dr. Hale Sezer |  |
| 12:15-13:00 | **LUNCH** | |  |
| 13:00-13:30 | Educator Roles and Characteristics | Prof. Dr. Hatice Şahin |  |
| 13.30-13.50 | Program Development in Interprofessional Education | Prof. Dr. Hatice Şahin |  |
| 13.50-14.10 | **COFFEE BREAK** | |  |
| 14.10-14.30 | Teaching Methods and Strategies | Prof. Dr. Seçil Arslansoyu Çamlar |  |
| 14.30-15.00 | Training Activities in Large Groups - Effective Presentation Skills |  |  |
| 15.00-15.30 | Learner-Centered Approaches |  |  |
| 15.30-16.00 | End-of-Day Feedback and Closing | Prof. Dr. Hatice Şahin – Assoc. Prof. Dr. Aysel Başer – Dr. Mustafa Küçük |  |
| **22.11.2024** | **BASIC TRAINER DEVELOPMENT - DAY 2** | |  |
|  | **LECTURE** | **INSTRUCTOR** |  |
| 09:00-09:45 | Opening, Day 1 Evaluation, Gathering Expectations, and Introduction of Day 2 Program | Assoc. Prof. Dr. Aysel Başer |  |
| 09.45-10.15 | Introduction to Assessment and Evaluation | Assoc. Prof. Dr. Funda İfakat Tengiz |  |
|  | Purpose and Types of Assessment and Evaluation |  |  |
| 10.15-10.45 | Preparing Multiple-Choice Questions and Structured Exams  (Designing valid multiple-choice questions and applying peer-reviewed multiple-choice question technical analysis.) | Assoc. Prof. Dr. Aysel Başer |  |
| 10.45-11.00 | **COFFEE BREAK** | |  |
| 11.00-11.30 | Small Group and Skills Training Activities, Coaching | Assoc. Prof. Dr. Hakan Gülmez |  |
| 11.30-12.00 | The Use of Technology in Teaching  (Interactive lesson demonstration with examples and applications in both large and small group teaching settings) | Assoc. Prof. Dr. Funda İfakat Tengiz |  |
| 12:00-13:00 | **LUNCH** | |  |
| 13.00-14.15 | Scenario-Based Application Example  (A practical application was conducted using tsunami and flood disaster scenarios, which are common disaster types in Tanzania) | Prof. Dr. Hatice Şahin – Assoc. Prof. Dr. Aysel Başer |  |
| 14.15-14.30 | **COFFEE BREAK** | |  |
| 14.30-15.30 | Program Evaluation  (Participants conducted a hands-on evaluation of the designed program through applied assessment methods.) | Assoc. Prof. Dr. Hale Sezer |  |
| 15.30-16.00 | End-of-Day Feedback and Closing | Prof. Dr. Hatice Şahin – Assoc. Prof. Dr. Aysel Başer |  |

Supplementary Table: Introduction to Disaster and Emergency Response (ADG +) Training of Trainers
